# Does trumpet playing affect lung function?—A case-control study

**DOI:** 10.1371/journal.pone.0215781

**Published:** 2019-05-02

**Authors:** Lia Studer, Desiree M. Schumann, Aline Stalder-Siebeneichler, Michael Tamm, Daiana Stolz

**Affiliations:** Clinic of Pulmonary Medicine and Pulmonary Cell Research, University Hospital Basel, Basel, Switzerland; Telethon Institute for Child Health Research, AUSTRALIA

## Abstract

**Objectives:**

The effect a wind instrument has on lung function is a much-discussed topic with inconclusive data, not least because existing studies combine all wind instruments as one entity. The aim of this study was to investigate the effect of playing a trumpet/cornet/flugelhorn on lung function.

**Methods:**

A prospective, multicentre, cross-sectional, case-control study. Participants were recruited from wind orchestra or brass bands throughout Switzerland. Inclusion criteria: aged 16 or older, reporting at least one year of playing a trumpet/cornet/flugelhorn. Controls were members of an orchestra, who had never played a wind instrument. The primary end-point of the study was the difference in FEV_1_%predicted between trumpet/cornet/flugelhorn players and controls.

**Results:**

147 subjects were included in the study. Controls (n = 48) were significantly younger, more commonly male, current smokers and had a lower body mass index compared to trumpet/cornet/flugelhorn players (n = 99). There was no significant difference in FEV_1_%predicted (91.9% vs 94.2%; p = 0.316) or FVC %predicted (89.4% vs 92.6%; p = 0.125) between controls and trumpet/cornet/flugelhorn players, respectively, in crude and adjusted analyses. However, there was a significant negative association between the number of years playing a trumpet/cornet/flugelhorn and FVC %predicted after adjusting for smoking status, gender, and height. Trumpet/cornet/flugelhorn players had a similar amount of respiratory symptoms to controls (questionnaire score 3.2±3.2 vs 2.8±2.4, p = 0.717).

**Conclusion:**

Lung function in trumpet/cornet/flugelhorn players was similar to controls. However, the number of years playing a trumpet/cornet/flugelhorn seems to have an adverse effect on forced vital capacity.

## Introduction

There is an ongoing discussion whether- and how- playing a wind instrument influences lung function. However, data provided are inconclusive [1–8]. Bouhuys *et al*. [1, 4] show that professional male wind instrument players have higher vital capacity (VC), total lung capacity (TLC) and forced expiratory volume in 0.75 seconds (FEV_0.75_) than healthy controls. Stauffer *et al*. [2] confirm that wind instrumentalists have higher VC than the established norms at that time. Sagdeo *et al*. [3] found a positive correlation between degree of expertise in playing a wind instrument and various lung function parameters such as forced vital capacity (FVC), forced expiratory flow between 25% and 75% of FVC (FEF25-75%), peak expiratory flow rate (PEFR), forced expiratory flow of 50% of FVC (FEF50%), forced expiratory flow of 75% of FVC (FEF75%) and maximum voluntary ventilation (MVV). Kim *et al*.[9] found an improvement in FVC, forced expiratory volume in 1 second (FEV_1_) and MVV in elderly women trained for 5 or 10 weeks in playing the ocarina, a closed wind instrument. Both Sagdeo *et al*.[3] and Kim *et al*.[9] use lung function in litres and not percent predicted. Conversely, Navratil *et al*. [5], Fuhrmann *et al*. [8], Schorr-Lesnick *et al*. [10], Smith *et al*. [11], Fiz *et al*. [12], Ksinopoulou *et al*. [13], Borgia *et al* [14], Brzek *et al*. [15] and Bouros *et al*. [16] could not establish any difference in lung function between wind instrumentalists and controls. Deniz *et al*. [17] report that playing a wind instrument is detrimental to lung function and Zuskin *et al*. [18] demonstrate a higher prevalence of sinusitis, nasal secretion and hoarseness in wind instrument players compared to controls. The possible detrimental effect of playing a wind instrument could be due to high-pressure damage to small airways and alveoli, or infection caused by pathogens residing in the instruments [8, 17, 19, 20]. Despite the scarce evidence of its beneficial effect, playing a wind instrument, such as a didgeridoo, is currently recommended as a treatment modality to increase awareness and compliance in asthmatics [21, 22]. A recent review concludes that due to the paucity of reliable data, only a weak recommendation can be made for music therapy in asthmatics [23].

Remarkably, most studies so far combine brass wind and woodwind instruments even though each instrument needs a very specific blowing technique [24–26]. Trumpets, cornets and flugelhorn are high brass instruments, in which players have to create the highest amount of pressure to produce a sound [6, 12]. Woodwind instruments include the flute, saxophone, and bassoon amongst others. While woodwind instrumentalists only have to blow correctly into their mouthpiece, brass wind players have to produce the sound by vibrating their lips [24]. It is tempting to postulate that the differences in the blowing-technique, amount of pressure needed to be generated and particularities of the distinct instrument classes could explain, at least partly, the contrasting results of the existing studies. Further possibilities for the differences in existing studies could be 1) selection bias–Stauffer et al [2] performed their experiments in U.S. Navy members who would need to be in good form based on their occupation; and 2) effects of smoking—Akgun and Ozgonul [4] had significantly more smokers in the research group compared to the controls. Deniz *et al*. [17] (n = 18 brass players and 16 woodwind players) and Navratil *et al*. [5] (n = 84 wind instrument players, it does not specify) found no difference in lung function between wood wind and brass wind instrumentalists but their sample size was relatively small.

The main objective of this cross-sectional, case-control study was to assess lung function in proficient trumpet/cornet/fluegelhorn players. We hypothesized that FEV_1_% predicted is higher in trumpet/cornet/fluegelhorn players than in controls since respiratory breathing exercises have shown positive effects in healthy and diseased individuals [9, 27, 28].

## Material and methods

### Study design and subjects

This was a prospective, cross-sectional, case-control study including an opportunity sampling provided from different regions of Switzerland. As it was an opportunity sampling, to fulfil the sample size required, we were unable to do an age and sex-matched study. Inclusion criteria for trumpet/cornet/fluegelhorn players were: a) playing either trumpet, cornet or flugelhorn in a wind orchestra or brass band; b) reported playing history of ≥1 year; c) reported playing time ≥ 2 hours per week. The inclusion criteria for controls were: a) playing any musical instrument other than a wind instrument in an orchestra or brass band;. Both trumpet/cornet/flugelhorn players and controls were required to be over 16 years old, not pregnant, depict proficiency in German allowing the subject to respond to a questionnaire and have the ability to provide informed consent.

Exclusion criteria of both groups were: a) chronic lung disease except for COPD or asthma; and b) respiratory tract infection symptoms within the last week.

### Protocol

This study was approved by the responsible ethics committee, Ethikkommission Nordwest- und Zentralschweiz (EKNZ 2017–00766). All participants signed a written consent before inclusion in the study. For patients under 18 years of age the informed consent was signed by a parent or legal guardian. The study was performed according to the Good Clinical Practice guidelines and the Declaration of Helsinki.

Medical technical assistants employed by the Clinic of Pneumology, University Hospital of Basel performed all measurements under the supervision of an intern and an attending pulmonologist. Lung function was measured with a portable EasyOne spirometer (New Diagnostic Design, Zurich, Switzerland) according to the ATS/ERS guidelines [29].

FeNO measurements were performed with the NIOX VERO (Circassia, Bad Homburg, Germany) according to the ATS/ERS recommendations for standardized procedures for the measurement of FeNO [30]. Participants were required to exhale into the instrument maintaining a constant pressure for 10 seconds. Before the measurements, participants completed a shortened version of the validated SAPALDIA 1 Questionnaire [31] to assess respiratory symptoms. The questionnaire consisted of 45 questions investigating symptoms such as wheezing, coughing, sputum, and dyspnoe, history of chronic lung diseases, comorbidities, exposure to environmental factors, and smoking status.

### Outcomes

The primary end-point of the study was the difference in FEV_1_%predicted between trumpet/cornet/fluegelhorn players and controls. Secondary outcomes included spirometry values such as FVC (in litres and %predicted), FEV1/FVC index and the fractionated exhaled nitric oxide (FeNO in ppb) as well as respiratory symptoms assessed by a questionnaire.

### Sample size calculation

With a significance of 0.005, a power of 90% and an unbalanced sample size ratio 2:1 (trumpet/cornet/fluegelhorn players:controls), a minimum sample size of 92 trumpet/cornet/fluegelhorn players and 46 controls was necessary to evidence a difference of 5% with a standard deviation of 7.2 [4].

### Statistical methods

Differences in dichotomous variables were evaluated using the Chi-square test or Fischer’s exact test, as appropriate. Normally distributed parameters were analyzed using the Student’s t-test for equality of means. All other continuously non-normally distributed parameters were evaluated using the non-parametric Mann-Whitney U test or Kruskal-Wallis test, as appropriate. When analysing the respiratory symptom questionnaire, the answers marked ‘not relevant’, ‘don’t want to answer’, ‘don’t know’ and ‘no’ were marked as zero, and the answer ‘yes’ as 1. The maximum possible score was 40 and the minimal possible score was 0. Higher scores indicate a higher number or severity of symptoms.

## Results

Between June 2017 and December 2017, 99 trumpet/cornet/fluegelhorn players and 48 controls were included in the study totalling 147 participants from more than 25 different orchestras in Switzerland. Of the 99 trumpet/cornet/fluegelhorn players, 44 played cornet only, 2 played cornet and fluegelhorn, 1 played cornet and trumpet, 6 played fluegelhorn, 1 played fluegelhorn and trumpet, 43 played trumpet only, and 2 played all three instruments. Controls were significantly younger, more commonly current smokers, male and presented a lower body mass index compared to the trumpet/cornet/fluegelhorn players (Table 1). The trumpet/cornet/fluegelhorn players played on average 5.2±4.1 hours per week, had played their instrument for an average of 27±14.4 years and cleaned their instruments once a year.

**Table 1:** Demographics of the high brass instrument players and control groups.

	High brass players (n = 99)	Controls (n = 48)	P-value
Age, in years SD	40.5±16.0	33.3±14.7	**0.008**
Male, n(%)	66 (67)	41 (85)	**0.018**
**Smoking status**			0.433
Current smoker	13 (13)	10 (21)	
Non-smoker	71 (72)	30 (63)	
Ex-smoker	15 (15)	8 (17)	
Body mass index, kg/m^2^	25.3±3.9	24.0±3.4	**0.037**
Current Medication*	30 (32.3)	12 (25.5)	0.412
History of asthma	2	1	0.980
Duration of instrument playing, in years	27±14.4	n/a	
Playing time per week, in hours	5.2±4.1	n/a	

*Data missing. Current medication included medication for hypertension, proton-pump inhibitors, anti-coagulants, etc. Continuous data are shown as mean±SD and categorical variables as number (%). FEV_1_ = forced expiratory volume in 1s, FVC = forced vital capacity, Tiffeneau-Pinelli index = FEV_1_/FVC; FeNO = fractional exhaled nitric oxide

### Lung function

There was no significant difference in FEV_1_%predicted or FVC % predicted between the trumpet/cornet/fluegelhorn players and controls using a Mann-Whitney U-Test (Table 2). Contrariwise, both absolute FEV_1_ and FVC were higher in controls, evidencing the demographic diversity of the two groups. FEV_1_/FVC, a marker of airflow limitation, lay in the normal range and did not differ between trumpet/cornet/fluegelhorn players (81.7±5.9) and controls (83.6±6.0). There was also no association between FEV_1_/FVC and trumpet/cornet/fluegelhorn players or controls using linear regression and adjusting for age, gender, smoking status and BMI.

**Table 2:** Comparing lung function parameters of the high brass instrument players and control groups using Mann-Whitney U-Test.

	Trumpet/cornet/fluegelhorn players n = 99	Controls n = 48	P-value
FEV_1_%predicted, in %	94.2±13.3	91.9±11.7	0.316
FEV_1_, in (L)	3.5±0.7	3.9±0.7	**0.004**
FVC %predicted, in %	92.6±11.7	89.4±11.2	0.125
FVC, in (L)	4.3±0.8	4.7±0.8	**0.007**
FEV1/FVC, in %	81.7±5.9	83.6±6.0	0.115

Abbreviations: FEV_1_ = Forced expiratory volume in 1sec.; FVC = forced vital capacity

**Fig 1:**
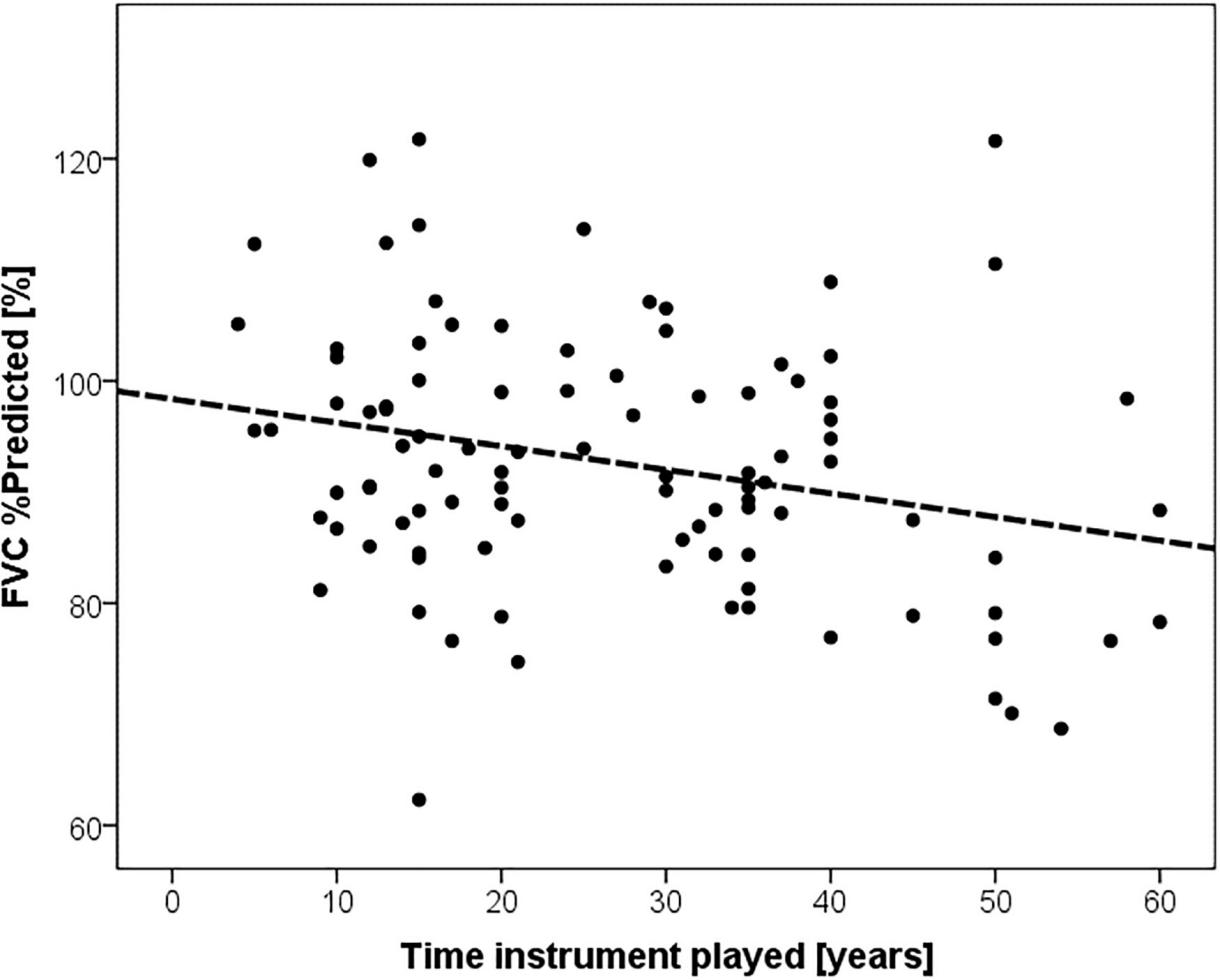
Number of years played was negatively associated with FVC %predicted, Beta = -0.265, p = 0.008. Eosinophilic airway inflammation.

There was a significant negative association between number of years playing a trumpet/cornet/fluegelhorn instrument and both the FVC %predicted (Beta = -0.265, p = 0.008) and the absolute FVC in L (Beta = -0.290, p = 0.029) after adjusting for smoking status, age, gender, and height (Linear regression, Fig 1). Importantly, despite the differences between the groups, all values were in the normal range.

Exhaled FeNO was similar in trumpet/cornet/fluegelhorn players and controls (18.9 ppm ±10.9 vs 23.4 ppm ±18.0, p = 0.278, respectively; Mann-Whitney U-test).

### Respiratory symptoms

There was no significant difference in respiratory symptoms between controls and trumpet/cornet/fluegelhorn players (2.8±2.4 vs 3.2±3.2, respectively, p = 0.717, Mann-Whitney U-test).

## Discussion

Uncertainty surrounding the impact of playing a wind instrument on lung function has been ongoing for several years. Various studies combined brass wind and woodwind instrumentalists, hampering the interpretation of results due to the differences in technique and pressure required when playing these instruments [2, 4, 8]. In the present study, the largest of its kind, we therefore, investigated the effect of playing a trumpet/cornet/flugelhorn, a homogenous group of wind instruments, on lung function compared to non-wind instrument playing controls who were also musicians in a brass band.

We found no significant difference in FEV_1_%predicted and FVC %predicted between trumpet/cornet/flugelhorn players and controls. There was also no significant difference in the FEV_1_/FVC index between trumpet/cornet/flugelhorn players and controls. Interestingly, there was a negative association between the number of years played and FVC (absolute and %predicted) even though the FVC was still in the normal range. It has been postulated that repeated deep inhalations, and increased pressure may damage alveoli and small airways [8, 17]. We, however, found no difference in respiratory symptoms between the trumpet/cornet/flugelhorn players and controls. The average age of the trumpet/cornet/flugelhorn players in our study was 40.5 years with an average duration of playing the instrument of 27 years. As the trumpet/cornet/flugelhorn players were relatively young, it was possible that continued playing could cause a continued decrease in FVC that could lead to a restrictive respiratory disease. A longitudinal study would be required to further evaluate the possible restrictive effect of playing a trumpet, cornet or flugelhorn on lung function. Although we were unable to analyse the microbial environment in the instruments, considering that the players only cleaned their instruments a median of once a year, a high probability existed that pathogenic microbes may have resided in the instrument which could have caused airway irritation or worse as has been shown before [19, 20, 32]. Although Granell et al. [33] found an association between playing a wind instrument and airway obstruction, we had found no difference in FeNO between trumpet/cornet/flugelhorn players and controls. The lack of symptoms and lack of eosinophilic inflammation did not negate the effect of playing trumpet/cornet/flugelhorn on FVC over time as the changes were still within normal range at this time point.

The main limitation of our study was that the trumpet/cornet/flugelhorn players and controls were not age-matched and the gender distribution differed. However, adjusted analyses were expected to account for these imbalances. Also, the careful selection of controls as instrument players with a similar life-style as the trumpet/cornet/flugelhorn players, was expected to generate a well-matched control group for phytosociological factors difficult to adjust for. The strengths of this study were the rather large, homogenous group of trumpet/cornet/flugelhorn players originating from several different orchestras and thus assuring the generalizability of the results. In addition to lung function, eosinophilic airway inflammation and respiratory symptoms were assessed.

In conclusion, there was no difference in FEV1% predicted between trumpet/cornet/flugelhorn players and controls. Nevertheless, in trumpet/cornet/flugelhorn players the time of exposure to instrument playing was associated with a decrease in forced vital capacity. Thus, caution may be required in recommending playing a trumpet/cornet/flugelhorn without long-term dedicated data from longitudinal studies evaluating healthy subjects and those suffering from respiratory illnesses.
